# A microscale biomimetic platform for generation and electro-mechanical stimulation of 3D cardiac microtissues

**DOI:** 10.1063/1.5037968

**Published:** 2018-10-29

**Authors:** Roberta Visone, Giuseppe Talò, Paola Occhetta, Daniela Cruz-Moreira, Silvia Lopa, Omar Antonio Pappalardo, Alberto Redaelli, Matteo Moretti, Marco Rasponi

**Affiliations:** 1 Department of Electronics, Information and Bioengineering, Politecnico di Milano, 20133 Milan, Italy; 2 Cell and Tissue Engineering Lab, IRCCS Istituto Ortopedico Galeazzi, 20161 Milan, Italy; 3 Department of Biomedicine, University Hospital Basel, 4056 Basel, Switzerland; 4 Regenerative Medicine Technologies Lab, Ente Ospedaliero Cantonale (EOC), 6900 Lugano, Switzerland; 5 Swiss Institute of Regenerative Medicine (SIRM), 6807 Taverne, Switzerland; 6 Cardiocentro Ticino Foundation, 6900 Lugano, Switzerland

## Abstract

Organs-on-chip technology has recently emerged as a promising tool to generate advanced cardiac tissue *in vitro* models, by recapitulating key physiological cues of the native myocardium. Biochemical, mechanical, and electrical stimuli have been investigated and demonstrated to enhance the maturation of cardiac constructs. However, the combined application of such stimulations on 3D organized constructs within a microfluidic platform was not yet achieved. For this purpose, we developed an innovative microbioreactor designed to provide a uniform electric field and cyclic uniaxial strains to 3D cardiac microtissues, recapitulating the complex electro-mechanical environment of the heart. The platform encompasses a compartment to confine and culture cell-laden hydrogels, a pressure-actuated chamber to apply a cyclic uniaxial stretch to microtissues, and stainless-steel electrodes to accurately regulate the electric field. The platform was exploited to investigate the effect of two different electrical stimulation patterns on cardiac microtissues from neonatal rat cardiomyocytes: a controlled electric field [5 V/cm, or low voltage (LV)] and a controlled current density [74.4 mA/cm^2^, or high voltage (HV)]. Our results demonstrated that LV stimulation enhanced the beating properties of the microtissues. By fully exploiting the platform, we combined the LV electrical stimulation with a physiologic mechanical stretch (10% strain) to recapitulate the key cues of the native cardiac microenvironment. The proposed microbioreactor represents an innovative tool to culture improved miniaturized cardiac tissue models for basic research studies on heart physiopathology and for drug screening.

## INTRODUCTION

Unraveling the mechanisms involved in cell differentiation and tissue generation and maturation remains an open challenge in the field of tissue engineering (TE). Organs-on-chip technology has recently emerged as a powerful tool to recreate tissue-specific physiological microenvironments not achievable with conventional 2D or 3D cultures.^1,2^ The ability to mimic *in vitro* the native tissue milieus and precisely control the microenvironment has been proved as key requirements to regulate cell behaviors and enhance cell function.^3,4^ Furthermore, miniaturization offers several advantages in biology, such as a significant reduction in the use of reagents and cells, allowing for increased experimental throughput in a cost-effective manner.^5,6^ By exploiting this technology, great effort has been made to develop robust *in vitro* cardiac models to provide better insights into heart physiology and pathology in an economically sustainable way.^7,8^

Biomimetic strategies aimed at providing biochemical and biophysical stimuli specifically tailored to fully recapitulate key aspects of the complex heterogeneous signals of the local heart tissue “niche” have been demonstrated to effectively guide tissue development and maturation.^9–12^ The myocardium is a highly organized 3D tissue in which mechanical forces together with electrical cues play a key role in promoting the cardiac function.^10^ In particular, cardiomyocytes (CMs) *in vivo* contract in a coordinated fashion, generating a continuous cyclic mechanical stretch.^13–15^ This deformation activates mechanosensitive signaling pathways, which positively affect CM properties and functionality.^16,17^ Moreover, the complex cell-cell interconnections through gap junctions ensure CM coordinated contraction, whose rhythm is maintained by pacemaker cells.^18^ An ideal cardiac *in vitro* model for developmental biology and drug discovery should present (i) 3D cell-cell and cell-extracellular matrix (ECM) interactions and organization and (ii) a biomimetic controlled mechanical and (iii) electrical stimulation.^19^

Microfluidic platforms have been deployed to generate functional cardiac microtissues with a 3D organization,^20^ by exploiting the spontaneous cell-cell connections or encapsulating cells within hydrogels.^11,21,22^ Cardiomyocytes derived from induced pluripotent stem cells (iPS-CMs) forming embryoid bodies^21^ or aligned microtissues^12^ have been successfully seeded and cultured within microfluidic platforms able to precisely deliver biochemical stimulation. Although microtissues were successfully generated, the cardiac models resulted in immature, lacking important structures (e.g., mature sarcomeres and intercalated disks), likely due to the poor reproduction of typical mechanical and electrical native cues,^13,20,23,24^ thus limiting their use for drug screening purposes. The application of a mechanical strain has been demonstrated to enhance cell organization, maturation, and functionality,^9,25^ highlighting the connections between cell mechanics and cell phenotype^26^ and between cell organization and performances.^27,28^ In the attempt to generate more complex microtissues, pairs of predefined structures (e.g., cantilevers and posts) were exploited as tools to drive the organization of cells embedded in hydrogels, allowing reproduction of the anisotropic structural orientation of native cardiac myofibers.^11,25,29,30^ Moreover, by adjusting the spring constant of the anchoring structures, the system allowed providing cells with an auxotonic load, reproducing the mechanical cues of the cardiac environment. However, dedicated channels for medium perfusion were not included in the platforms, impairing a fine control over the biochemical conditioning. To merge the advantages of microfluidic platforms with the potentiality of enhancing tissue properties by means of mechanical stimulation, our group developed for the first time an innovative microfluidic platform providing cardiac microtissues with a cyclic mechanical deformation, mimicking the contraction-relaxation phases experienced by cells *in vivo.*^9^ The mechanical training of cardiac cells resulted in a superior well-defined geometry and mechanical and electrical properties of the microengineered cardiac tissues with respect to statically cultured microtissues, which were suitable for drug screening purposes. Furthermore, by exploiting microfluidic channels, a fine control over biochemical stimulation was also possible.

On the other hand, electrical stimulation has been proven to play a pivotal role, alone or together with mechanical and biochemical stimulation, in ameliorating the structural and functional properties of the engineered myocardium.^31–33^ Therefore, several strategies have also been adopted to combine electrical and mechanical stimulations in a single platform.^9,11,33–37^ In particular, electrical systems were designed to either guide cardiac microtissue maturation or test tissue functionality. These works have some limitations as follows: (i) a non-uniform electric field on the cell constructs, due to the lack of *ad hoc* integrated electrical systems,^9,25^ and (ii) the lack of consensus about an optimal electrical stimulation protocol for the generation of mature cardiac models, able to improve the spontaneous and synchronous cardiac beating.^9,33,34^ Indeed, electrical stimulation of cardiac cells has been pursued by either controlling the external electric field or the flowing current. In particular, the most used stimulation patterns have aimed at imposing either an electric field intensity *E* of 5 V/cm (2 ms, 1 Hz)^31^ or a current density *j* of 74.4 mA/cm^2^ (2 ms, 1 Hz).^38^

Despite great advances in the organs-on-chip technologies, none of the micro-physiological systems currently developed was able to fully recapitulate the complexity of the cardiac native milieu. Here, we present an innovative microfluidic platform designed to provide 3D cardiac microtissues with a controlled biomimetic environment for biochemical, electrical, and mechanical conditioning: lateral medium channels allow for a biochemical conditioning, an integrated electrical system provides a tightly controlled electric field, and a pressurized chamber ensures a mechanical stretching of the microtissues. The previously described beating heart on the chip platform^9^ was thus equipped with an innovative electrical system compatible with the microscale nature of the device and its peculiar mechanism of mechanical stimulation. In this study, we exploited the new microfluidic platform to investigate the effect of the two previously mentioned electrical stimulation approaches (*E *=* *5 V/cm and *j *=* *74.4 mA/cm^2^)^31,38^ on 3D cardiac microtissues obtained from neonatal rat cardiomyocytes. Furthermore, as proof of concept, we studied the effect of the combined application of physiological cyclic uniaxial strain (10%–15%) and electrical stimuli.

## RESULTS

### Design and fabrication of the microfluidic platform

The microfluidic platform design consists of three different functional elements: (i) a culture chamber for 3D cell-laden hydrogel culture, (ii) a pressure-actuated compartment providing a cyclic mechanical stretching to the microtissues by exploiting a previously described technique,^9^ and (iii) a pair of stainless-steel electrodes.

**FIG. 1.**
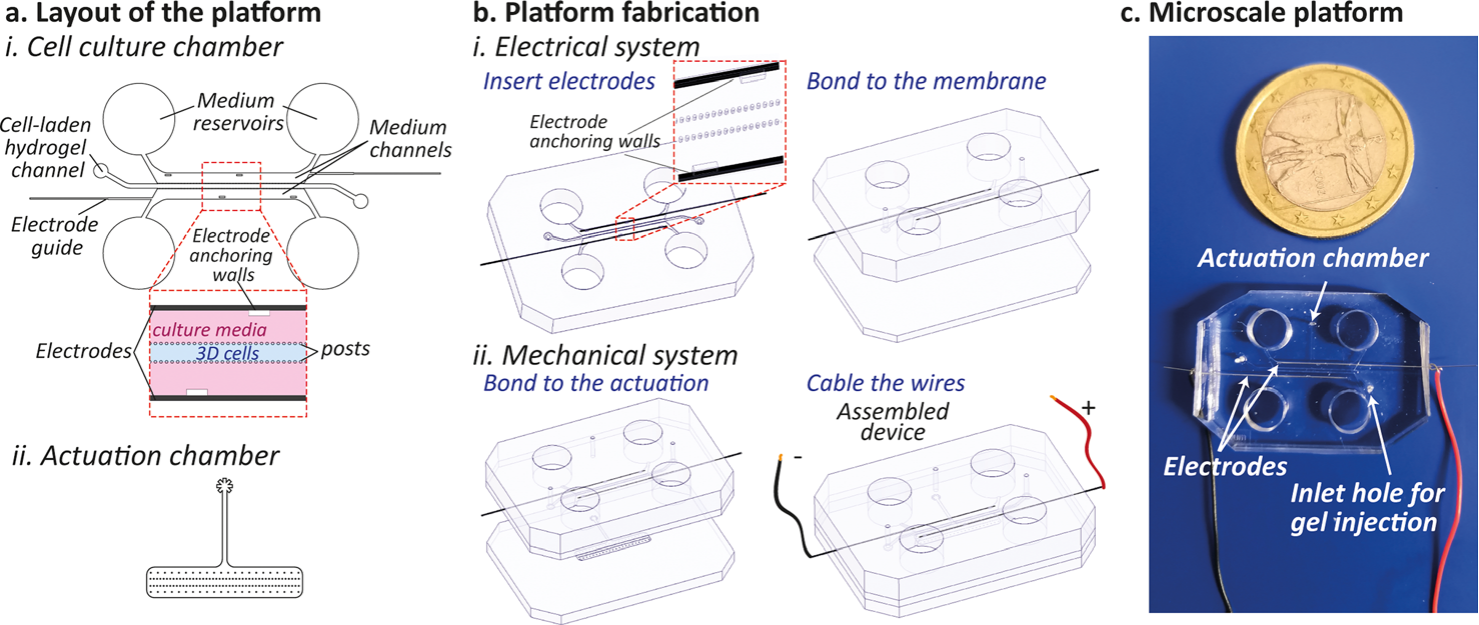
(a) Layout of the microfluidic device depicting (i) the cell culture chamber and (ii) the pressure actuated compartment. (b) Detailed microfluidic platform fabrication steps encompassing the electrode insertion, the bonding of the cell culture layer to the PDMS membrane, and the implementation of the mechanical system by including the pressure-actuated chamber. (c) Photograph of the final assembled PDMS microscale platform.

The cell culture chamber [Fig. 1(a-i)] is composed of three channels, defined by two rows of hanging posts with a hexagonal cross-section.^9^ The central channel hosts the cell-laden hydrogel during culture, while the lateral channels and reservoirs furnish the culture medium to the microtissue. Stainless steel electrodes are placed in the lateral medium channels and precisely positioned parallel to the main axes of the microtissues through specific guides and anchoring walls. The pressure actuated compartment [Fig. 1(a-ii)] consists of a rectangular chamber containing 4 rows of circular posts.

The entire platform was produced by assembling multiple layers of polydimethylsiloxane (PDMS), obtained through soft-lithography techniques. The two compartments are separated by a thin membrane: the application of a positive pressure in the actuation chamber causes the stretching of the microtissues,^9^ while the elastic recoil of PDMS allows to gain its rest position when pressure is released. The location of the electrode pair [Fig. 1(b)] does not perturb the mechanical actuation system, which ensures a uniaxial strain to the cells of about 12%, as fully described previously.^9^ As shown in the picture of Fig. 1(c), the final microscale platform was equipped with four reservoirs for the cell culture medium, two ports for cell injection, one port for pressure control, and two copper wires for electrical stimulation.

### Electrical characterization of the platform

**FIG. 2.**
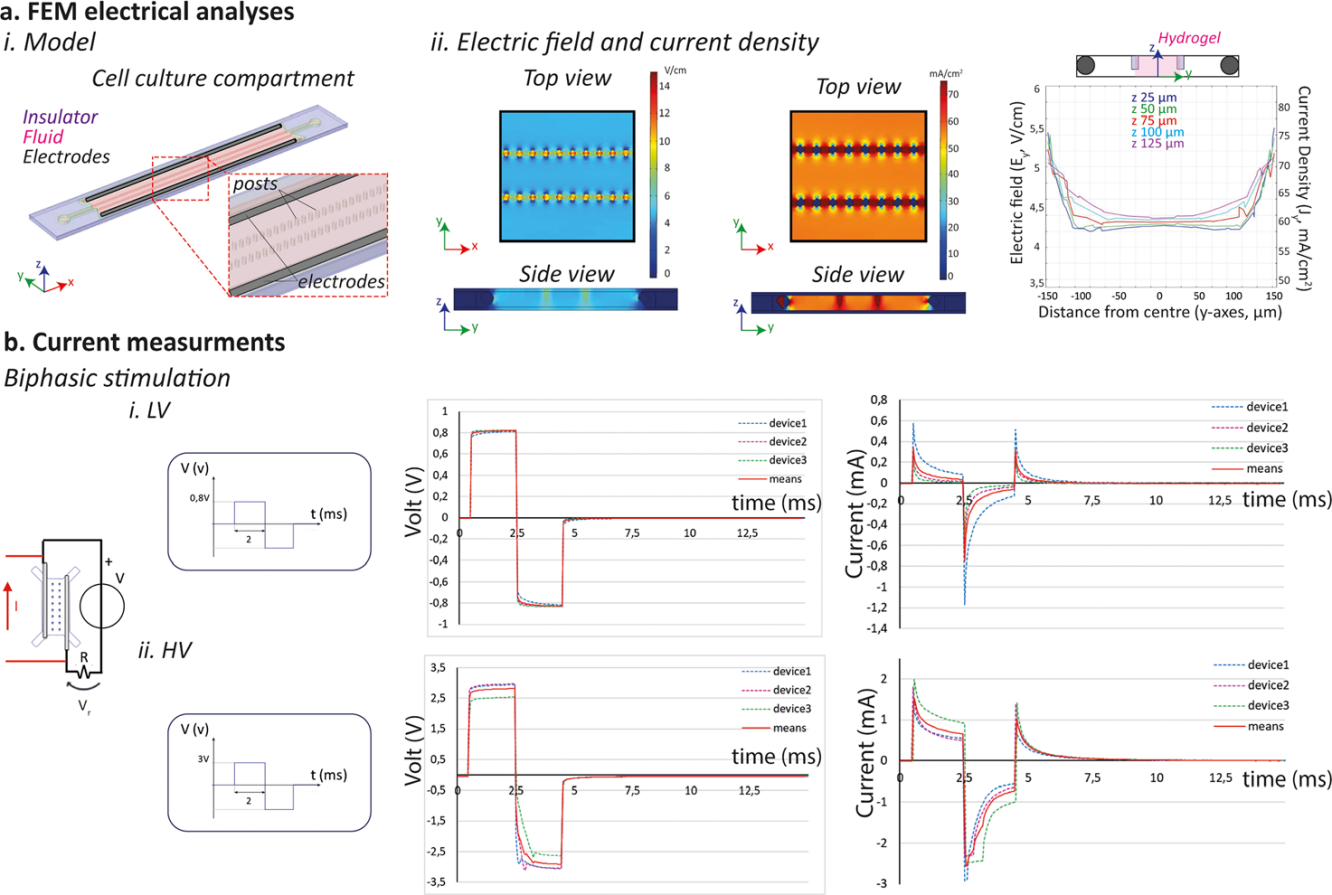
(a) FEM electrical analyses performed (i) by modeling the cell culture chamber which comprised the PDMS structure (insulator), the culture medium (fluid), and the electrode (electrodes). (ii) Uniformity of the electric field and current density achieved within the platform. (b) Electrical characterization of the device: experimental setup used to provide the device with a biphasic electrical stimulation of (i) 5 V/cm electric field (LV) and (ii) 74.4 mA/cm^2^ current density (HV), with the correspondent curves of the measured voltage drop and calculated current within the platform. The values of n = 3 devices were plotted, with the correspondent mean.

Finite element methods (FEMs) were used to verify electric field and current density uniformity provided within the platform by the parallel configuration of the electrodes. The geometrical domain was modeled as follows: the culture medium as a conductive material (σ = 1.5 S/m and ε_r_ = 80.1), the electrodes as a highly conductive material (σ = 1.32 × 10^6^ S/m and ε_r_ = 1.005), the PDMS hanging posts as good insulators (σ = 10^−22^ S/m and ε_r_ = 2.63), and the culture compartment walls as perfect electrical insulators [Fig. 2(a-i)]. As shown in Fig. 2(a-ii), the electric field became uniform in the region occupied by the microtissue, with values around 4.3 V/cm for the entire height of the chamber, when 5 V/cm was applied. Either higher (5.5 V/cm) or lower (4 V/cm) values were computed in the lateral regions, corresponding to the gaps between or underneath posts or nearby the posts themselves, respectively. However, as evidenced in the plot of Fig. 2(a-ii), the electric field values remain rather constant throughout the height of the central channel region.

Similar results were obtained for the current density distribution [Fig. 2(a-ii)]. A uniform value for the current density (about 60 mA/cm^2^) was achieved in the inner part of the cell culture channel. In the outer regions, values ranging between 75 mA/cm^2^ and 35 mA/cm^2^ were due to the presence of hanging posts. In the gaps underneath the confinement structures, the values of the current density increased, coherently with the electric field trend. However, also in this case, the values of the current density remained substantially constant throughout the total height of the central channel region.

An experimental characterization of the electrical system was performed to assess the actual current flowing within the microfluidic platform. In particular, two different tests were designed, controlling either the electric field (positive peak value 5 V/cm) or the current density (positive peak value 74.4 mA/cm^2^), by means of biphasic rectangular waves with 2 ms duration and 1 Hz frequency, as suggested for cardiac cell stimulation.^32^

Owing to geometrical considerations (electrode distance of 1.6 mm), an electric potential of 0.8 V was applied on the external wires to achieve an electric field intensity *E *=* *5 V/cm within the platform. Conversely, an electric potential of 3 V on the electrodes was estimated which is necessary to provide a current density *j *=* *74.4 mA/cm^2^. Thus, the electrical stimulation patterns used will be referred from now on as Low Voltage (LV) and High Voltage (HV) stimulations.

The biphasic stimulations highlighted the complex electrical behavior of the device as a resistor-capacitor circuit, as evidenced by the exponential trend of the voltage drop and current curves depicted in Fig. 2(b).

During the LV stimulation, the positive and negative values of the voltages exponentially reached 0.8 V and −0.8 V, as expected. The current intensities displayed a positive peak corresponding to the on/off events of the stimulus (mean values are 0.35 mA and 0.32 mA, respectively) and a higher negative peak in correspondence to the inversion of voltage polarity (mean value, −0.75 mA). The average positive and negative plateau values were obtained to be 0.03 mA and −0.06 mA, respectively.

Similar results were achieved during HV stimulation. Voltage drops rose to average positive and negative values of 2.8 V and −2.9 V, respectively, with exponential trends. Coherently with LV, the current intensity reached the positive peak of 1.5 mA before setting to a plateau value of 0.65 mA and a negative peak of −2.5 mA before setting to −0.73 mA. The positive peak due to the inversion of signal polarity rose to 1 mA. Moreover, in all tested conditions, the time constant of the system was lower than 2 ms (duration of the applied stimulus), demonstrating the suitability of our system in providing an efficient electrical stimulation.

### Electrical stimulation of cardiac microtissues

**FIG. 3.**
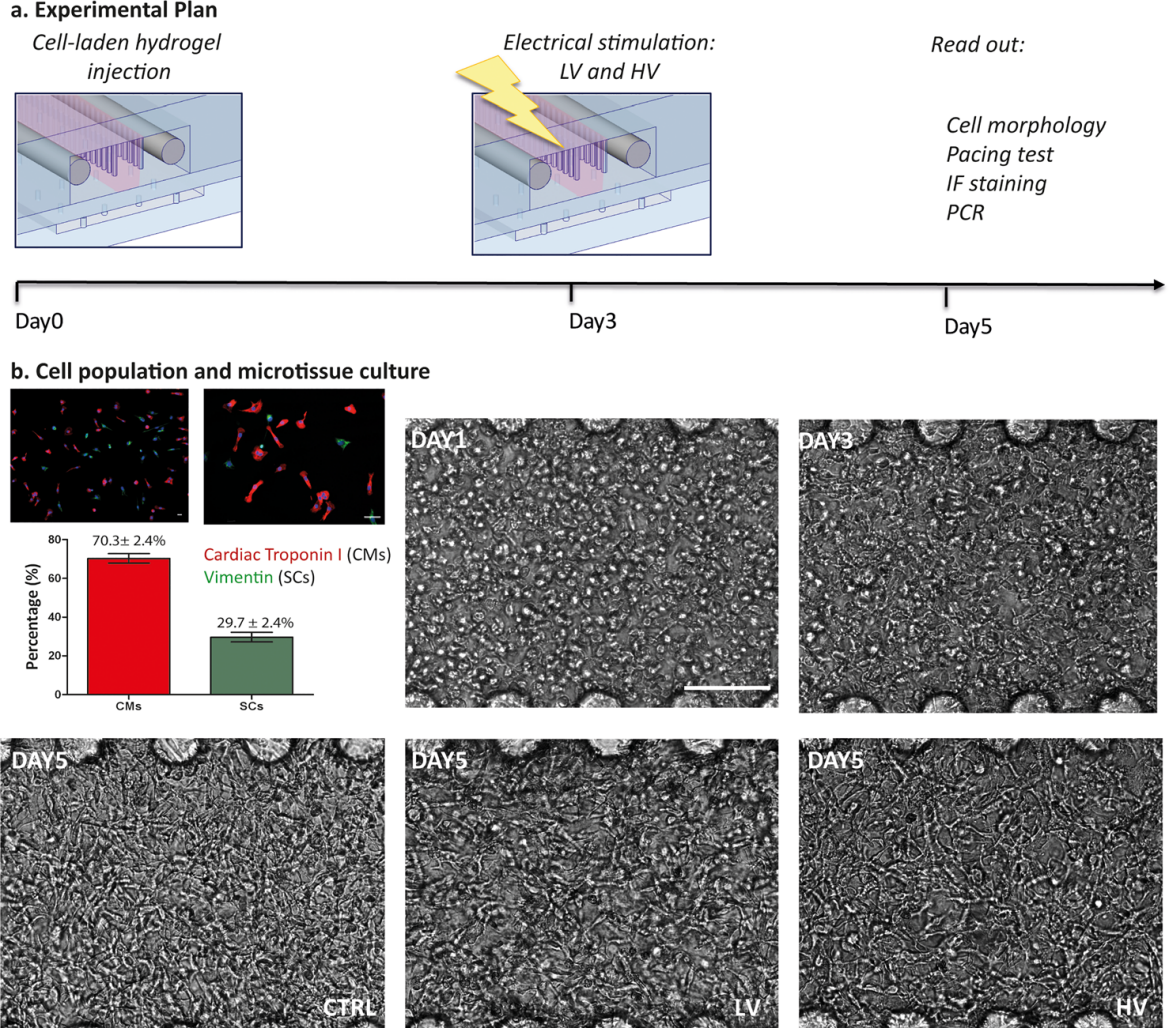
(a) Experimental plan showing that cells are seeded in the devices, electrically stimulated after 3 days of static culture and analyzed at day 5. (b) The initial cell population is composed of cardiomyocytes (70.3% ± 2.4%) and cardiac stromal cells (29.7% ± 2.4%) as quantified from immunofluorescence images. Once embedded in fibrin gel and cultured in the platform, they start elongating and connecting. The scale bar is 100 *μ*m.

To assess the effect of the electrical stimulation, cardiac microtissues were statically cultured within the platform for 3 days and then electrically stimulated for 2 other days, as depicted in Fig. 3(a). The initial cell population used to generate the cardiac microtissues was mainly composed of cardiomyocytes (70.3% ± 2.4%), identified by the cardiac Troponin I and by a smaller fraction of cardiac stromal cells (29.7% ± 2.4%), stained through vimentin [Fig. 3(b)].

After seeding, cells were monitored to evaluate the development of the microtissues, as shown in Fig. 3(b). In detail, the day after seeding cells appeared mostly round, with some initial sprouting. Already at day 3, they started elongating and forming connections with neighboring cells, and at day 5, they assumed an elongated shape and an intrinsic high cell-cell interconnection within microtissues in all conditions [Fig. 3(b)].

**FIG. 4.**
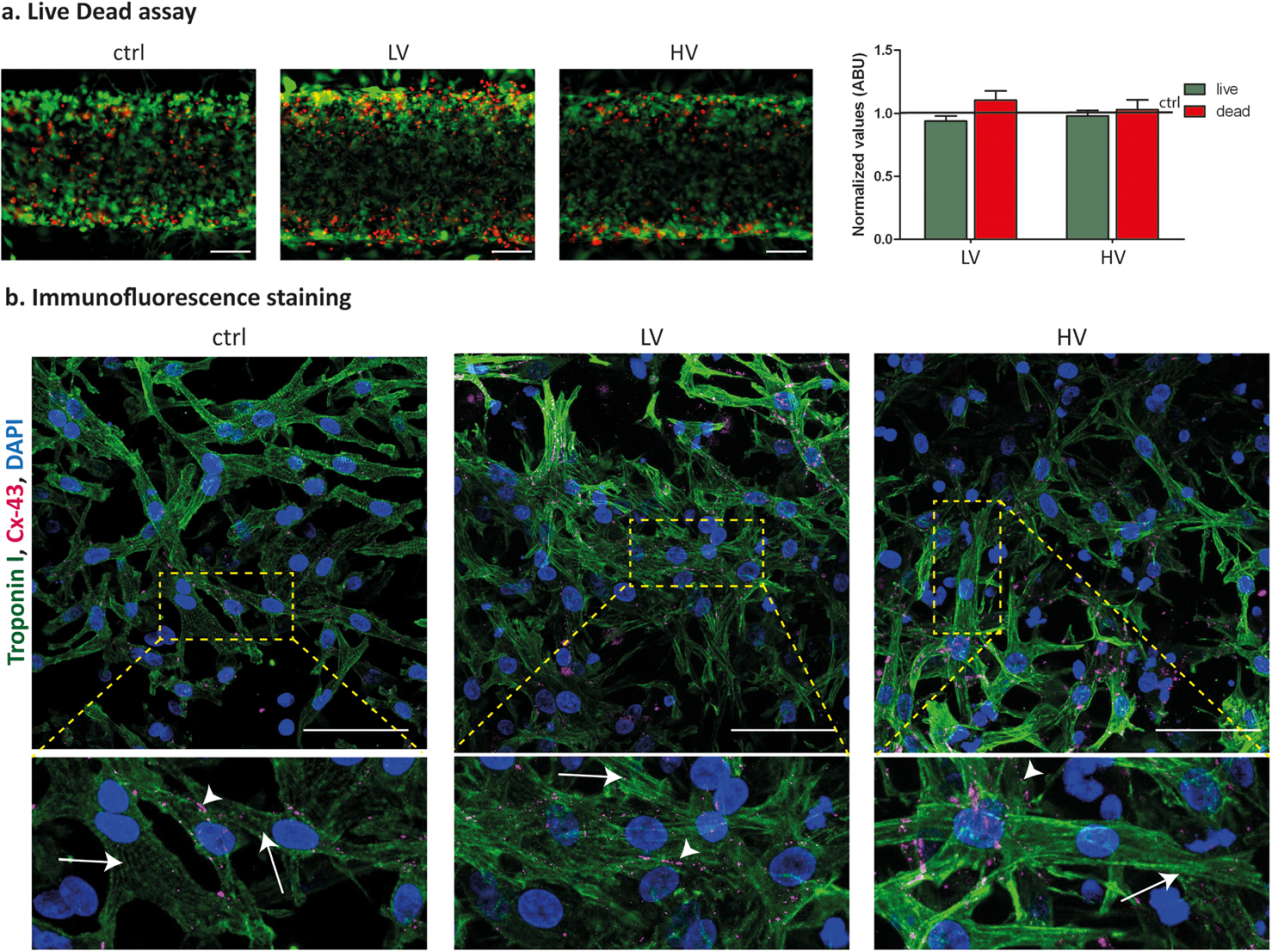
(a) Live and dead staining and quantification, evidencing similar cell viability in both control and electrically stimulated microtissues after 5 days in culture. (b) Immunofluorescent staining of cardiac Troponin I and Connexin-43, highlighting the typical rod-like shape of cardiomyocytes and the expression of electrical gap-junctions after 5 days in culture. Arrows indicate the cell striation and arrow heads the gap junctions at the membrane. The scale bar is 100 *μ*m.

In Fig. 4(a), the majority of the cells were alive after 5 days in culture, as evidenced by the Calcein AM and ethidium staining, and no statistical differences were estimated between the two provided electrical stimulations since the percentage of live cells was obtained to be 91.9% ± 0.05% and 98.1% ± 0.04% for the LV and HV, respectively. A slightly higher concentration of cardiomyocytes was observed nearby the posts [Fig. 4(a)].

Cardiac Troponin I immunofluorescence staining in Fig. 4(b) evidences that CM showed the typical elongated and rod-like shape in both electrically stimulated and control conditions, as well as visible cytoskeletal striations.

**FIG. 5.**
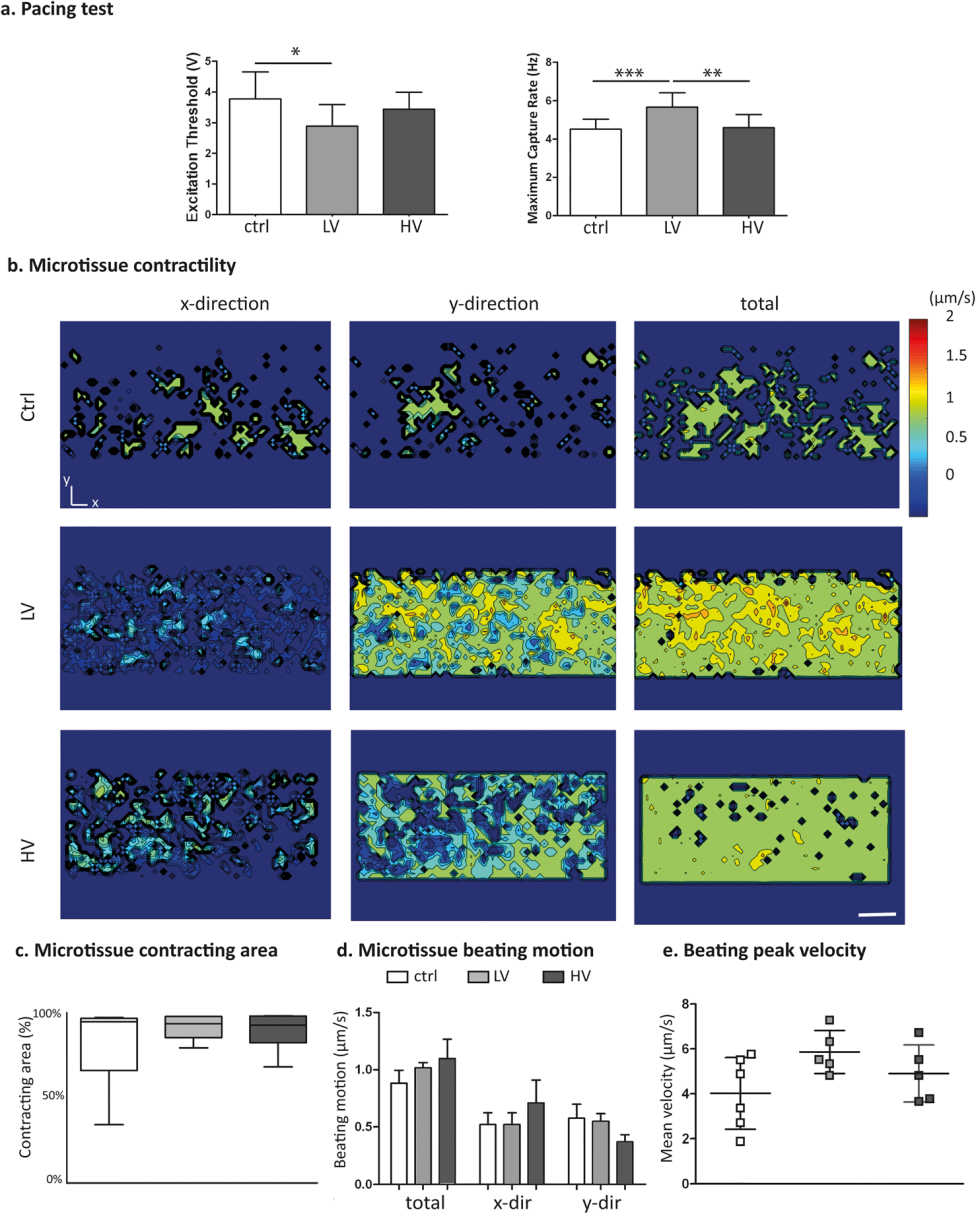
(a) Pacing tests performed to assess the ET and MCR parameters, indicating microtissue electrical excitability. (b) Representative color maps of the time-averaged beating motions of the control and electrically stimulated microtissues after 5 days in culture, paced at 1 Hz. The scale bar is 100 *μ*m. (c) Percentage of the area of the microtissue involved in the contraction and (d) directionality of the beating. (e) Microtissue beating peak velocities.

These findings were supported by the functional evaluation of the microtissues, which was assessed by analyzing beating parameters upon pacing tests [Fig. 5(a)]. In particular, the Excitation Threshold (ET), defined as the minimum voltage to induce microtissue synchronous beating with a pacing signal, and the Maximum Capture Rate (MCR), the maximum frequency at which the microtissues synchronously beat following the pacing, were evaluated. Both electrically stimulated microtissues showed a lower ET (2.9 ± 0.7 V and 3.4 ± 0.5 V for the LV and HV stimulations, respectively) as compared to the control group (3.8 ± 0.87 V), evidencing a higher cell excitability. Notably, differences in the ET value from LV and control microtissues were statistically significant, demonstrating a higher electrical connection between cells due to stimulation. The superior electrical coupling achieved was also confirmed by the MCR parameter, which was statistically enhanced in LV stimulation (5.67 ± 0.74 Hz) as compared to both HV (4.6 ± 0.6 Hz) and control (4.5 ± 0.5 Hz). In Fig. 5(b), the analyses on the beating of microtissues during pacing at 1 Hz revealed an enhanced percentage of the contracting area in stimulated tissues, without an apparently preferential orientation [Fig. 5(c)]. Furthermore, the peak velocity during beating [Fig. 5(d)] increased for LV microtissues (5.8 ± 0.9 *μ*m/s), as compared to HV (4.9 ± 1.2 *μ*m/s) and control (4 ± 1.5 *μ*m/s). Calcium transient analyses revealed a synchronized beating of the construct in all conditions. Moreover, in both LV and HV microtissues, the calcium transient amplitude was higher with respect to the controls, whose values were 3-fold lower (SI 3).

Cardiac microtissues were preliminary tested for the response to isoprenaline, a non-selective β-adrenoreceptor agonist commonly used to treat bradycardia. In Fig. SI 4(a), it is evidenced how the spontaneous beating frequency of cardiac microtissues increased in a dose dependent manner in all the condition tested. Moreover, the contraction velocity in both spontaneously beating and paced constructs seemed to increase at high isoprenaline concentrations (from 10 nM to 1000 nM), for control and LV conditions. Such a trend was less evident in the HV condition [Figs. SI 4(b) and SI 4(c)].

### Electro-mechanical stimulation of cardiac microtissues

**FIG. 6.**
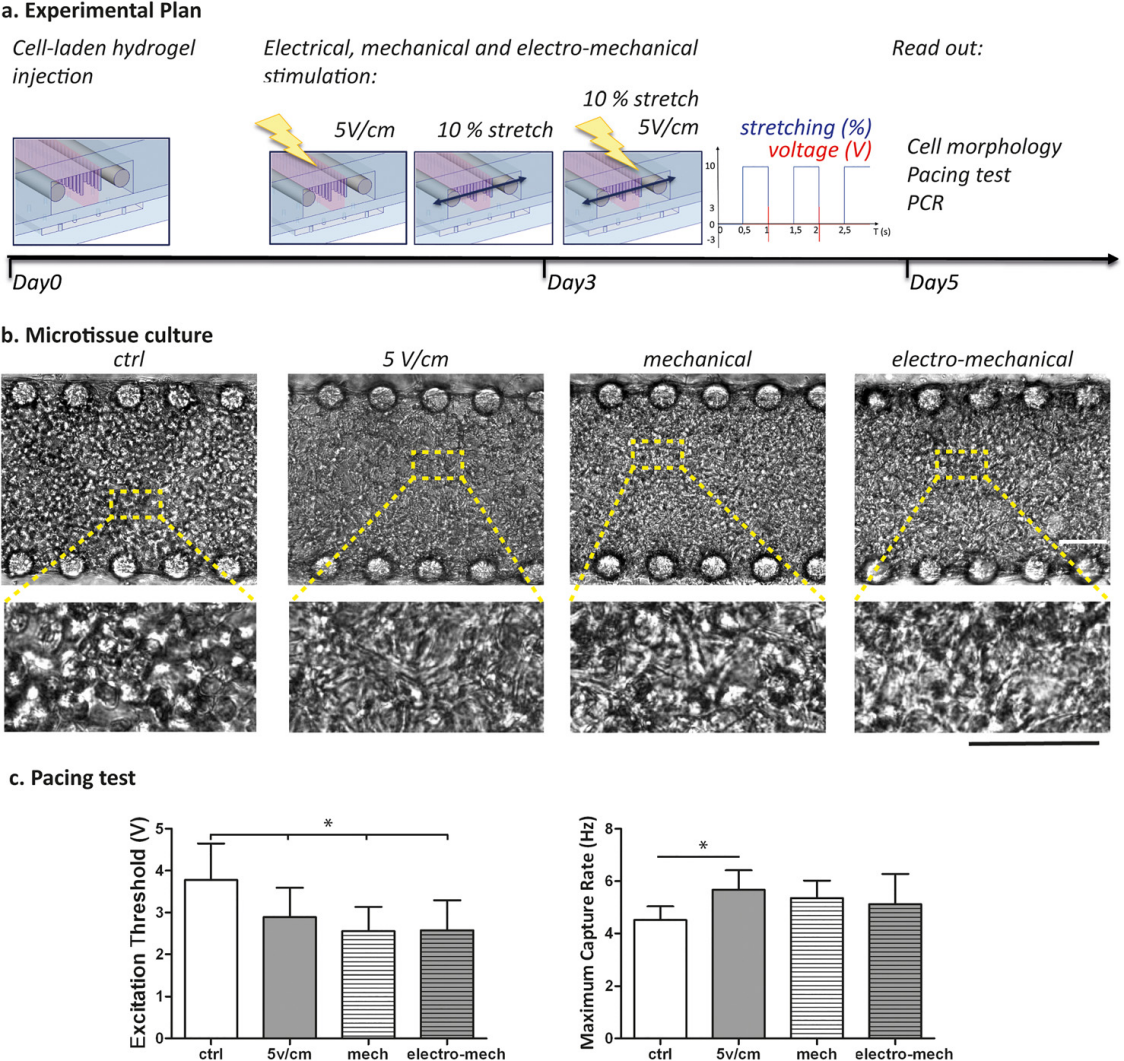
(a) Experimental plan showing that after seeding, cells are statically cultured for 3 days, and then the electrical (5 V/cm), mechanical (10% stretch, 1 Hz), or electro-mechanical stimulations were started. At day 5, all the analyses were performed. (b) Cell morphology assessed after 5 days of cultures, with higher magnification insets showing elongated cells. The scale bar is 100 *μ*m. (c) Pacing tests performed to evaluate microtissue electrical excitability, through ET and MCR parameters.

Both mechanical^9^ and electrical stimulations independently demonstrated to enhance cardiac microtissue functionalities within our system. Therefore, as proof of concept, to better mimic the *in vivo* cardiac environment, we combined the previously described mechanical strain^9^ with the LV electrical stimulation. The mechanical stretching of 10% (500 ms duration, 1 Hz) and 5 V/cm electric field (2 ms duration, 1 Hz) were synchronized, as sketched in Fig. 6(a), to start the electrical stimuli (systole) when the mechanical actuation switches off (end of diastole).

Bright field images in Fig. 6(b) showed cell elongation and interconnections in all conditions after 2 days of stimulation (day 5 of culture), confirming previous findings. In particular, the cardiomyocyte morphology evidenced a better rod-like shape only in stimulated conditions (electrical, mechanical, and electro-mechanical) as compared to control.

Pacing tests [Fig. 6(c)] demonstrated an enhanced cardiac microtissue functionality achieved through electrical (5 V/cm, 1 Hz), mechanical (10% stretch, 1 Hz), or electro-mechanical stimulation. Indeed, ET was found to be statistically lower in all stimulated conditions, being 2.9 ± 0.7 V for electrically stimulated microtissues, 2.5 ± 0.5 V for mechanically stretched constructs, and 2.5 ± 0.7 for cardiac microtissues under electro-mechanical stimulation, as compared to control (3.7 ± 0.71 V). Although mechanical and electro-mechanical microtissues showed higher beating frequency as compared to control (5.3 ± 0.6 Hz, 5.1 ± 1.1 Hz, and 4.5 ± 0.5 Hz, respectively), only the electrical stimulation condition was found to generate statistically different MCR values (5.7 ± 0.7 Hz).

## DISCUSSION

The microfluidic platform presented in this study integrates all key-physical cues present in the native cardiac environment (i.e., biochemical, mechanical, and electrical). The enhancement of the maturation level of cardiac microtissues represents a strict requirement for the generation of robust cardiac *in vitro* models.^39^ Different strategies demonstrated that the mechanical and electrical properties of the myocardium promote cellular growth, orientation, maturation, and overall structural tissue organization.^4,9,16,33^

The active cyclic mechanical strain has been fully exploited in 2D systems, where cells were cultured on flat deformable membranes.^40,41^ However, the typical complex 3D organization of the cardiac tissues was not fully recapitulated, impairing the investigation of representative cell-cell or cell-matrix interactions. In the attempt to overcome these limitations, a technique relying on the self-organization of cell-embedded hydrogel microtissues for the generation of cardiac microwires (CMWs) was introduced by Legant *et al.*^30^ Briefly, cells were embedded in collagen or fibrin hydrogels and cast into PDMS molds containing flexible structures such as microscale cantilevers or micro-posts. During cell remodeling of the matrices, tissue collapsing and anchoring to the structures caused their deflection, proportional to cardiac microtissue development and beating,^11,25^ exerting in turn an auxotonic load to cells. This elegant strategy was also implemented in a 96-well device,^42^ demonstrating the potentiality of the system to generate functional human cardiac organoids for high-throughput studies in human biology and drug screening. In this contest, merging the concept of miniaturization with mechanical cues, our group recently developed a new biomimetic platform for culturing 3D fibrin-based cardiac microtissues, which enhances their maturation by means of active physiological cyclic mechanical strain.^9^ The key feature relied on the integration within a microfluidic device of a mechanical system, which allowed converting a pressure signal into a controlled uniform uniaxial strain applied to the 3D microtissues.

Besides mechanical cues, electrical stimulation has been largely exploited to develop cardiac tissues with enhanced conductive and contractile properties, leading to a better ultrastructural organization.^21,32,33,43^ The most commonly adopted engineering solutions to perform electrical stimulations are either carbon rods or platinum electrodes integrated into macroscale systems. Although efficient, these electrodes cannot be easily integrated within the microfluidic platforms, impairing the possibility to accurately control the uniformity of the generated electric field.^44^ Multi-electrodes arrays (MEAs)^45^ and other microelectrodes systems^46^ overcome these limitations, but their planar nature inhibits the achievement of uniform electrical stimulation in 3D microtissues. The first example of 3D electrodes integrated within microfluidic platforms was presented in a study by Pavesi *et al.*,^47,48^ in which a conductive PDMS/carbon nanotube mixture was cast into a silicone mold and successfully integrated within a microfluidic platform. However, the overall process became long and complex, and such geometry still did not allow to culture 3D cell microtissues.

Aiming at reproducing the complexity of the stimulation that cardiac cells experience *in vivo*, we here developed a biomimetic microfluidic platform, providing cells with a 3D environment in which biochemical, mechanical, and electrical features can be tailored to guide microtissue maturation. The platform relies on the integration of a couple of micro-stainless-steel electrodes that are directly and easily integrated within the microfluidic platform during the fabrication process. The design of the platform was maintained compatible with the previously optimized mechanical system, so as to equip the microfluidic device with both electrical and mechanical stimulations. The system was exploited to investigate the effect of electrical stimulation on cardiac microtissues, comparing the effect of two of the most effective strategies used at the macroscale to culture neonatal rat cardiomyocytes: applying a uniform electric field (i.e., 5 V/cm) or a uniform current density (i.e., 74.4 mA/cm^2^).

Since the electrical stimulation was the main feature added to the platform, a characterization of important electrical parameters was performed, while the mechanical characterization is fully detailed in our previous work.^9^ A FEM numerical model allowed us to confirm the generation of a uniform electric field over the hydrogel channel region with field perturbation only around the hanging post structures. The computational analyses were made by assuming steady state conditions, in agreement with previously reported studies,^38^ providing information about the spatial distribution of the electric field and current density. Moreover, to assess the dynamics of the involved electrical processes, an experimental characterization study was performed, highlighting the complex behavior of the system as a resistor-capacitor circuit. The electric current flowing in the device indeed reached a maximum value at the beginning of the pulse (positive or negative pulse) and then set to a plateau value. This is in line with previously reported works, evidencing complex phenomena occurring at the electrode-electrolyte interface associated with the use of solid electrodes in contact with the culture medium.^38,49,50^ The experimental characterization study allowed us to validate the stainless-steel electrode configuration as a suitable system to provide an efficient electrical stimulation within the microfluidic platform since the time constant of the system was lower than 2 ms (duration of the applied stimulus). Furthermore, it was possible to define the necessary voltage drop to achieve the pre-defined 74.4 mA/cm^2^ within the microfluidic platform, allowing for performing all cellular experiments with a voltage control stimulation, ensuring comparison between the electric field and current density conditions.

The platform was suitable to generate microtissues with well-defined dimensions, determined by the main channel geometry (300 *μ*m width, 150 *μ*m height, and 10 mm long), from neonatal rat cardiomyocytes. Both electrical stimulations were extremely low cytotoxic, confirming the suitability of the use of stainless-steel electrodes for electrical stimulation. The analyses on microtissue beating revealed that both electrical stimulations improved the responsiveness of the microtissues to an external pacing, as evidenced by the lower excitation threshold and higher maximum capture rate achieved. These results are in agreement with previously reported works both at the macro- and micro-scales.^32,51^ However, the application of the lower electric field (LV), defined as characteristic for the native myocardium,^32^ was more suitable to improve cell-cell coupling, statistically enhancing ET and MCR parameters, respect to control, and statistically increasing the MCR parameter compared to current density stimulation. Electrical stimulation increased cellular interconnections, enhancing the uniformity of the generated microtissues, as demonstrated by the higher percentage of the contracting area involved in the beating motion. Furthermore, the overall beating peak velocity was enhanced by electrical stimulation, as already shown for mechanical stimulation within our platform.^9^ The values of the beating velocity of microtissues achieved with electric field stimulation were comparable with previously reported data on the human cardiac construct cultured in a 3D environment within a micro-physiological platform,^12^ even if a predominant beating direction was not evidenced in our system. Calcium transient analyses revealed a synchronized beating of the construct in all conditions. Moreover, in both LV and HV microtissues, the calcium transient amplitude was higher with respect to the controls, whose values were 3-fold lower (SI 3). These preliminary curves showed the compatibility and the potentiality of the platform to assess important parameters to characterize the calcium dynamics of 3D cardiac microtissues, such as the time to peak, the decay time, and peak height. Moreover, we preliminary exploited the platform to perform drug screening tests, by assessing the positive chronotropic effect of the isoprenaline on the cardiac constructs, as previously reported.^9^ Although variations of contraction velocity were observed based on drug concentration, further experiments are still required to better evaluate a dose-dependent effect.

To fully exploit the capabilities of the designed microfluidic platform as an innovative tool to perform biological studies, a pivotal experiment was performed to elucidate the effect of the combination of the electric field stimulation and cyclic mechanical deformation (electro-mechanical condition). In particular, starting the electrical pulse when the mechanical load is turned off mimics the *in vivo* isovolumic contraction of the heart.^52^ During a cardiac cycle, blood fills the ventricles, causing a stretch of the myocardium which is then electrically stimulated to contract (without relaxing) to generate the right pressure to pump blood through aortic or pulmonary valves. According to a study performed on cardiac ring-shape microtissues from fibrin gel stretched and subjected to electrical stimulations,^52^ the use of combined stimuli enhances the beating characteristics of the microtissues. Indeed, microtissues stimulated both electrically and mechanically showed statistically lower ET values as compared to control and were comparable with those achieved with either electrical or mechanical stimulation alone. However, the MCR did not reach values comparable to those in electric field stimulated microtissues, even if higher than the control. These results could be related to the fact that the electrical or mechanical stimulations provided alone were already optimized within the microfluidic platform, while the combination of the stimuli requires further investigation to induce optimal results.

## CONCLUSION

The developed microfluidic platform provides the unprecedented opportunity to precisely control different environmental cues (i.e., biochemical and physical) so as to generate 3D functional cardiac microtissues. The combination of uniform uniaxial strain and uniform electrical stimulation (either concurrently or sequentially) could be of fundamental relevance to improve the generation of more mature and robust *in vitro* cardiac models, especially envisioning the use of human induced pluripotent stem cell derived cardiomyocytes.^24,35^

## METHODS

### Ethics statement

In this study, cells were isolated from hearts of 2 day-old neonatal Sprague Dawley rats (Charles River, Wilmington, MA, USA) that were collected from animals involved and euthanized in another study unrelated to the ongoing research. All the applicable international, national, and/or institutional guidelines for the use of these animals were followed. The Institutional Animal Care and Use Committee of the San Raffaele Scientific Institute (IACUC 795) approved the study designed for this group of animals. All procedures on animals involved in this study were in accordance with the ethical standards.

### Design and fabrication of the platform

The layout of the layers composing the platform was drawn using CAD software (AutoCAD, Autodesk Inc.). The central channel of the cell culture chamber is 300 *μ*m wide, delimited by hexagonal hanging posts. The media channels are 800 *μ*m wide and housed stainless steel electrodes (120 *μ*m in diameter) positioned at a distance of 1.6 mm. For each layer, the corresponding optical masks were obtained with a vector graphics editor (Adobe Illustrator CC, Adobe Systems Incorporated) and printed at high resolution (64 000 DPI) on a polyester film. Master molds were fabricated in a cleanroom environment (Polifab, Politecnico di Milano) by means of the conventional photolithography technique. Briefly, the pattern of each layer was transferred on SU8‐2050 photoresist (MicroChem, USA), previously spin-coated on 4 in. silicon wafers. The culture chamber master mold was composed by two layers of photoresist [Fig. SI 1(a)]: (i) a 50 *μ*m thin layer representing the cell culture chamber that forms the gap space between pillars and bottom compartment and (ii) a 100 *μ*m thick layer including the posts that were aligned on top of the previous culture chamber layer. Specifically, to obtain 50 *μ*m and 100 *μ*m photoresist layers, the spinning velocity used was 3840 rpm and 1900 rpm, respectively. The pressure actuated compartment is conversely obtained by a single photoresist layer, 50 *μ*m thick [Fig. SI 1(b)], obtained with a velocity of 3480 rpm.

The device was made of PDMS (Sylgard 184, Dow Corning) by mixing the elastomer base with the curing agent and degassing and casting it on the master molds. After curing (65 °C for 3 h), PDMS stamps were peeled off the mold and assembled, by permanently bonding surfaces with air plasma (Harrick Plasma, Inc.). In detail, holes for media reservoir and ports for hydrogel injection were created within the cell culture chamber through biopsy punchers of 5 mm and 750 *μ*m, respectively. Stainless steel electrodes were manually positioned in the stamp which was subsequently bonded to a PDMS membrane (1 mm thick). The access port for the pressure-actuated compartment was then created by punching a hole of 500 *μ*m, and the so-obtained layer was bonded to the pressure-actuated compartment, after careful alignment. Electric wires were soldered by means of epoxy conductive resin (RS Components) to the electrodes. The final assembled device was further cured overnight at 80 °C to finalize the bonding process and wire connections.

### Finite element analyses

FEM was performed through commercially available software (COMSOL Multiphysics 4.2a, Stockholm, Sweden). The geometry characterizing the cell culture chamber [Fig. 2(a)] was divided into three domains: (i) the fluid domain, representing the culture medium, (ii) the electrodes, and (iii) the insulator comprising the PDMS frame and posts. For the medium, electrodes, and PDMS domain, the electrical conductivity of 1.5 S/m, 1.32 × 10^6^ S/m, and 10^−22^ S/m and the relative permittivity of 80.1, 1.005, and 2.63 were defined.^43^ Numerical simulations were performed exploiting the *electric current* interface, assuming DC and steady state conditions to solve Maxwell's equations.^38,49^ Insulation boundary conditions were applied to PDMS surfaces; stainless steel electrodes were set to ground and 0.8 V to simulate the application of a 5 V/cm electric field. The electric field and the current density distribution were visualized by plotting colorimetric maps, while the accurate evaluation of the intensity of the generated electric field and current density was investigated by plotting the 1D graph at different quotes, corresponding to the different height of the microtissues.

### Functional characterization of the electrical mechanism

Electrical signals for the electrical characterization of the platform were generated and recorded through the analogical channels of the data acquisition (DAQ) board (NI USB-6211, National Instrument, TX, USA), by providing monophasic waves (2 ms, 1 Hz), which were set up through a custom-made LabVIEW interface (LabVIEW, National Instrument, TX, USA). The microfluidic platform was connected in series to a 150 Ω resistor. Voltage drop on both the microfluidic platform and the resistor was acquired, and the current flowing in the circuit was calculated by using the Ohm law on the resistor. Two electric potentials were applied to the circuit, reproducing an electric field of 5 V/cm or a current density of 74.4 mA/cm^2^. In the first case, an electric potential of 0.8 V was applied across the electrodes, whose distance is 1.6 mm. In the second case, a preliminary test was performed to obtain the voltage drop on the platform which ensures a current density of 74.4 mA/cm^2^. In detail, a current of 1 mA was calculated to be necessary flow through the platform, whose cross-section was estimated to be 1.4 mm^2^. This current was thus applied through the platform, and the corresponding voltage drop of 3 V was acquired. For each device, results were plotted as means of 10 s registration with a sample rate of 20̇000 samples/s. For each test, the average values of voltage drops and of the currents in the devices were also plotted.

### Development of a custom-made electro-mechanical stimulator

A custom-made control system based on LabView was developed, to interconnect a multipurpose DAQ-MX I/O board with the microfluidic devices [Fig. SI 2(a)]. A current amplifier circuit, made of four Op-Amp in buffer configuration and 8 bipolar junction transistors, was assembled to increase the output current of the I/O board (limited to ±2mA per channel), of both positive and negative signals.

In particular, the system allowed generating two individually tunable analog square waves with amplitudes ranging from ±0.001 V to ±10 V and frequencies from 0.01 Hz to 1 kHz. For the electrical stimulation, biphasic square waves were generated with 0.4% duty cycle (2 ms positive voltage and 2 ms negative voltage and 996 ms off). For the pacing tests, monophasic square waves with 0.4% duty cycle (4 ms on and 996 ms off) were generated. In addition, a transistor-transistor logic (TTL) port of the board was used to actuate a solenoid miniaturized valve connected to the mechanical actuation compartment o the microfluidic platform.^9^ The LabView software allowed us to independently control the analog and digital signals so as to synchronize the electro-mechanical stimulation of cardiac microtissues. The graphical user interface was conceived to set stimulation parameters, such as the signal amplitude and the frequency, and to capture and plot the platform's electrical response in real time and to save the data [Fig. SI 2(b)].

### Cardiac cell isolation and characterization

Neonatal rat cardiomyocytes were isolated from 2-days-old Sprague Dawley rats following the previously described protocol.^9^ Freshly isolated cells were enriched for cardiomyocytes by performing a 1 h pre-plating in flasks. The harvested non-adhered cells, which were mostly cardiomyocytes, were cultured in Dulbecco's modified essential medium high glucose (DMEM, 4.5 g/l glucose, Gibco) supplemented with 10% v/v fetal bovine serum (FBS, Hyclone), 100 U/ml penicillin (Life Technologies), 100 *μ*g/ml streptomycin (Life Technologies), and 10 mM Hepes (Gibco), defined as the growth medium. Cells were used the day after to generate the cardiac microtissues. To characterize the initial cell population, cells were also seeded in monolayer at a density of 8 × 10^4^ cells/cm^2^ on 0.1% w/v gelatin (Sigma Aldrich) coated wells. Neonatal rat cardiomyocytes and stromal cells were immunofluorescence stained for cardiac Troponin I (cTnT) and vimentin (Abcam), respectively. Image analysis was exploited to calculate the relative percentage of the two cell populations by normalizing the values over the total amount of cells, quantified by the 4',6-diamidino-2-phenylindole (DAPI, Invitrogen) nuclear staining.

### Cardiac microtissue culture and stimulation

To generate the 3D cardiac microtissues, cardiomyocytes were embedded in a fibrin gel at a density of 75 × 10^6^ cells/ml and were injected into the microfluidic platform. In detail, a fibrinogen solution was mixed with the cardiac cells suspended in DMEM containing thrombin and aprotinin, in order to achieve a final fibrin gel of 20 mg/ml fibrinogen, 5 unit/ml of thrombin, and 1.6 TIU/ml of aprotinin (all purchased from Sigma Aldrich). The so-obtained cell-laden fibrin solution was pipetted into the central channel of the culture chamber (∼2 *μ*l each device) and incubated for 5 min (5% CO_2_; 37 °C) to allow the gel reticulation before adding the culture medium in the lateral channel. The microtissues were cultured for 5 days in the growth medium supplemented with 2 mg/ml of aminocaproic acid (Sigma). Both types of electrical biphasic stimulations (fixed electric field of 5 V/cm and constant current density of 74.4 mA/cm^2^) were applied after 3 days in culture. Static cultures of cardiac microtissues were used as control.

The most suitable electrical stimulation conditions were subsequently exploited to evaluate the effects of the combined electro-mechanical stimulation. In particular, after 5 days in static culture, mechanical (mech, 10% uniaxial strain, frequency 1 Hz), electrical, or combined (electro-mech) stimulations were provided to microtissues after 3 days in culture. Static cultures of cardiac microtissues were used as control.

### Cell viability

To evaluate the cytocompatibility of the microfluidic device and to prove the non-cytotoxic effect of the provided electrical stimulations, cell viability was assessed on both stimulated and control microtissues after 5 days in culture. The live/dead assay (Thermo Fisher Scientific) was performed according to the manufacturer's protocol. Briefly, the microtissues were washed twice with pre-warmed PBS, and then, a solution of 2 *μ*M Calcein AM and 4 *μ*M Ethidium homodimer in phosphate buffered saline (PBS) was perfused within the devices. After 15 minutes of incubation at 37 °C in the dark, images were acquired by means of a fluorescence microscope (Olympus IX-71, Olympus Corporation, Tokyo, Japan) at 10× magnification. Green-labeled live cells and red-stained nuclei of dead cells were quantified using ImageJ software, and results were expressed as normalized values respect to the control condition. Three samples for each condition were considered.

### Immunofluorescence staining

Immunofluorescence analyses were performed after 5 days of culture on electrically stimulated microtissues directly within the microdevices to assess cardiac phenotype, maturation (Troponin I), and electrical coupling (Connexin-43). Samples were fixed in 4% paraformaldehyde (PFA) for 15 min and incubated with a solution of 0.1% Triton X-100 (Thermo Fisher Scientific) and 2% bovine serum albumin (BSA, Sigma Aldrich) in PBS for 1 h at room temperature to permeabilize cells and to block nonspecific binding. Primary antibodies, Troponin I (Santa Cruz) and Connexin-43 (Abcam), were diluted 1:100 and 1:500, respectively, in 0.5% w/v BSA and incubated overnight at 4 °C. Goat anti-mouse (Thermo Fisher Scientific, rhodamine conjugated) and goat anti-rabbit (Thermo Fisher Scientific, FITC conjugated) secondary antibodies, diluted 1:200 in 0.5% w/v BSA, were incubated in the dark for 6 h at 4 °C. DAPI counterstaining was used to identify cell nuclei, by incubating the dye in PBS for 10 minutes at room temperature.

### Microtissue functional analyses

Electrical properties of microtissues were evaluated after 5 days in culture, performing functional pacing tests. The pacing signal was transferred to the microtissues by means of the integrated electrodes and consisted of a monophasic pulse of 4 ms duration, generated through the custom-made electrical stimulator, controlled via our Labview interface (Fig. SI 2). The amplitude (0–12 V, with 0.1 V resolution) and frequency (1–10 Hz) of the signal were adjusted for each microtissue to measure the excitation threshold (ET) and the maximum capture rate (MCR), as previously described.^9^ Data from 10 microtissues, from the N = 3 independent experiments, were analyzed.

Beating microtissues were monitored under a 10× objective lens on an inverted microscope (Olympus IX-71), and videos were collected through a camera at 23 frames/s to assess the contraction amplitude and beating synchronicity of electrically stimulated and control microtissues. The contraction amplitude and velocity were evaluated by means of a motion tracking software developed by Healy's group. Briefly, a region of interest (ROI) was defined for each microtissue, by excluding the regions nearby the posts. The area of the microtissues involved in the contraction, the mean contraction amplitude along x and y axes, and the mean beating peak velocity were assessed for 6 microtissues in the N = 2 independent experiments.

### Calcium transient assay

Assessments on calcium handling of cardiac microtissues in the LV, HV, and control conditions were performed after 5 days in culture. Oregon Green^®^488 dye (Thermo Fisher Scientific) at 10 *μ*M in the growth medium was injected in the media channels and incubated in the dark at 37 °C, 5% CO_2_ for 90 minutes. Fluorescence videos (Figs. SI 5–SI 7) were acquired using an inverted microscope (Olympus IX-71) equipped with a fast camera (100 frame/s) and were processed with ImageJ and Origin to generate fluorescence intensity plots. The peak amplitude, defined as the ratio between peak fluorescence intensity during contraction and baseline fluorescence (F/F_0_), was evaluated in both central and lateral parts (near posts) of the microtissues.

### Drug response

Dose–response experiments were performed on cardiac microtissues after 5 days in culture in LV, HV, and static conditions. Isoprenaline was diluted at different concentrations (0.001 nM, 0.1 nM, 10 nM, and 1 *μ*M) in the growth medium and added stepwise to the microtissues. After 15 minutes of incubation at 37 °C in a 5% CO_2_ environment, the response on the microtissues was recorded using an inverted microscope (Olympus IX-71), and videos were collected using a fast camera at 100 frame/s. The beating frequency and contraction velocity of both spontaneously beating and paced constructs (amplitude of 1.5-fold ET and frequency of 1 Hz) were evaluated by means of a motion tracking software developed by Healy's group. Results are presented as mean ± standard error for n = 3 microtissues.

### Statistical analyses

All data were statistically analyzed through GraphPad Prism software (GraphPad Software, USA). Data were tested for normality and presented and analyzed with mean ± standard deviation or median with quartile, accordingly. Statistical analyses were performed using Analysis of Variance (ANOVA) with Tukey's post-test or Kruskal-Wallis with the Dunn post-test (*P < 0.05, **P < 0.01, and ***P < 0.001).

## SUPPLEMENTARY MATERIAL

See supplementary material for the detailed steps to fabricate the microfluidic platform, the custom-made control system to perform the electro-mechanical stimulation, calcium transient assay, and drug response of microtissues. Supplementary videos of calcium transient are also available.
